# Validation of the Child version of the Perseverative Thinking Questionnaire of repetitive negative thinking in young people with diagnosed depressive and anxiety disorders

**DOI:** 10.1111/bjc.12336

**Published:** 2021-10-06

**Authors:** Caitlin Hitchcock, Renee L. Brown, Vanessa E. Cobham

**Affiliations:** ^1^ MRC Cognition and Brain Sciences Unit University of Cambridge UK; ^2^ Melbourne School of Psychological Sciences University of Melbourne UK; ^3^ School of Psychology The University of Queensland Brisbane Queensland Australia; ^4^ Children’s Health Queensland, Child and Youth Mental Health Service Brisbane Queensland Australia

**Keywords:** repetitive negative thinking, Perseverative Thinking Questionnaire, rumination, worry, validity, young people

## Abstract

This paper sought to provide the first validation of a transdiagnostic measure of repetitive negative thinking – the Perseverative Thinking Questionnaire‐Child version (PTQ‐C) – in young people diagnosed with anxiety and depressive disorders. Participants (*N* = 114) were 11‐ to 17‐year‐olds with complex and comorbid presentations seeking treatment through Child and Adolescent Mental Health Services. Confirmatory factor analyses best supported a three‐factor model for the PTQ‐C; however, hypotheses of both perfect and close fit were rejected, and a subsequent bifactor model suggested minimal unique variance for each subscale. Results demonstrated good internal consistency, convergent validity and divergent validity for the total score and three PTQ‐S subscales: core characteristics, perceived unproductiveness, and consumed mental capacity of negative repetitive thinking. PTQ‐C scores did not account for additional variance in anxiety symptoms once worry was considered, indicating that retention of a content‐specific measure may be warranted in clinical samples. Findings emphasize the importance of validating clinically relevant measures which were developed with subclinical populations in samples with diagnosed mental health disorders.

**Practitioner points:**

- Validates Perseverative Thinking Questionnaire in anxious and depressed youth.
- Support for convergent and divergent validity, and internal consistency.
- Results suggest measure is appropriate for complex and comorbid presentations.

## Background

Accurate identification of transdiagnostic cognitive processes which drive symptoms of multiple disorders is critical to enabling process‐focussed treatment of emotional disturbance. Repetitive negative thinking is a core feature of a range of anxiety and mood disturbances, along with sleep and substance use disturbances which frequently co‐occur with emotional disorders (McEvoy, Watson, Watkins, & Nathan, 2013). Traditionally, assessments of repetitive thinking have taken a content‐focussed rather than process‐focussed approach, focussing on identifying the features of repetitive thought in a disorder‐specific context. In the context of depression, rumination is defined as the process of thinking perseveratively about one’s feelings and problems (Nolen‐Hoeksema, Wisco, & Lyubomirsky, 2008). In the context of anxiety, worry is defined as seemingly uncontrollable chains of thoughts and images about a future event (Borkovec, Robinson, Pruzinsky, & Depree, 1983). More recently, the *process* of repetitive negative thinking has been recognized as more important in predicting comorbidity and prognosis than the disorder‐specific content associated with rumination and worry (e.g., Spinhoven, van Hemert, & Penninx, 2018). Process‐focussed assessment also comes with other advantages, as items on content‐specific measures of repetitive thinking are often confounded with items used for symptom measurement, limiting their utility for prospective risk assessment. Taking a process focus also allows truly transdiagnostic consideration of this risk factor for mental ill‐health. Here, we seek to validate a transdiagnostic measure of repetitive negative thinking for young people for the first time in a sample diagnosed with a variety of anxiety and depressive disorders.

There are currently two transdiagnostic self‐report measures that have been developed to assess repetitive negative thinking in young people, both of which have been previously validated in school‐based samples. The Repetitive Thinking Questionnaire‐10 (McEvoy, Mahoney, & Moulds, 2010) has been validated with 12‐ to 18‐year‐old adolescents, using the same items as the adult version of the assessment (McEvoy et al., 2019). The Perseverative Thinking Questionnaire‐Child version (PTQ‐C; Bijttebier, Raes, Vasey, Bastin, & Ehring, 2015) was adapted for a younger audience from the adult version of the PTQ, has been validated with a slightly younger age range (9–15 years), and therefore may be more suitably matched to attendees of Child and Adolescent Mental Health Services (CAMHS). Within the PTQ‐C, repetitive negative thinking is conceptualized as a process which is experienced as (1) repetitive, intrusive, and difficult to disengage from (defined as the ‘core characteristics’); (2) is perceived as unproductive; and (3) consumes mental capacity. The initial validation of the PTQ‐C in a community sample of 9‐ to 15‐year‐olds demonstrated adequate internal consistency, and convergent validity with other measures of worry, rumination, anxiety, and depression. Predictive validity was determined in a later prospective study (Bijttebier et al., 2018) with a community sample of 9‐ to 14‐year‐olds, with PTQ‐C scores found to longitudinally predict depressive symptoms. As in the adult version of the questionnaire, both one‐ and three‐factor (i.e., factors for core characteristics, perceived unproductiveness and mental capacity) models have indicated adequate fit in confirmatory factor analysis, with a one‐factor model being preferred for parsimony in both prior studies with young people (Bijttebier et al., 2015, 2018).

Critically, the validity and factor structure of the PTQ‐C has been determined in community samples of young people with subclinical symptoms. Individuals requiring treatment for anxiety and depression engage more frequently in repetitive negative thinking and experience the process as more uncontrollable and distressing than their community counterparts (Via et al., 2018; Yang et al., 2014; Yook, Kim, Suh, & Lee, 2010), such that repetitive negative thinking is a key target for transdiagnostic treatments (e.g., Unified protocol; Ehrenreich‐May et al., 2017). Validation of the PTQ‐C in young people with diagnosed anxiety and depressive disorders is thereby necessary to indicate its clinical utility and enable accurate assessment of a proposed mechanism of transdiagnostic psychological intervention. As transdiagnostic interventions are most frequently delivered to individuals with comorbid or co‐occurring disorders, validation with a sample experiencing one or more clinical disorders would ensure high ecological validity.

The current study therefore evaluated the PTQ‐C in a sample of youth experiencing diagnosed anxiety and depressive disorders. Specifically, we aimed to confirm the factor structure of the PTQ‐C, explore convergent and divergent validity, and relationships with symptoms of emotional disturbance in a treatment‐seeking CAMHS population with complex and comorbid presentations. If repetitive negative thinking is truly a transdiagnostic process in which assessment of context is unnecessary, content‐specific measures of worry and rumination would be expected to account for minimal variance in anxiety and depressive symptoms, respectively, once variance attributable to repetitive negative thinking had been removed.

## Methods

### Participants

The sample consisted of 114 young people (60% female) aged 11–17 years (*M* = 14.88, *SD* = 1.52). Completion of a simulation‐based power analysis (using pwrSEM; Wang & Rhemtulla, 2021) for the one‐factor model supported in the subclinical validation, estimating the factor loadings reported by Bijttebier et al. (2018), indicated that 114 participants would provide 90% power. Participants were recruited as a part of a randomized controlled trial of a transdiagnostic cognitive‐behavioural intervention for youth with severe and complex mental health presentations (trial pre‐registration: ACTRN12618000333213). Participants were recruited from six public‐system Child and Youth Mental Health Services in Brisbane, Australia. Referrals to the service are obtained through schools, external health professionals, or hospitals. Eligibility criteria for the trial (and thus this study) were that the youth was 11‐ to 18‐years‐old, presenting with a primary presentation of an anxiety or depressive disorder, able to engage with cognitively based therapy materials, and were able to engage in individual therapy. Exclusion criteria were intellectual disability and severe suicidality such that case management – rather than individual therapy – was the focus of service provision.

### Measures

#### Perseverative Thinking Questionnaire‐Child Version (PTQ‐C)

The PTQ‐C (Bijttebier et al., 2015) is a 15‐item self‐report measure of repetitive negative thinking. This scale was adapted for young people from the adult version created by Ehring et al. (2011). Items are scored on a 5‐point Likert scale from 0 (never) to 4 (almost always). Items are calculated to yield a total score, which can range from 0 to 60. The measure’s convergent, divergent, criterion, and predictive validity have previously been supported in non‐clinical samples of young people (Bijttebier et al., 2015, 2018).

#### Penn State Worry Questionnaire for Children (PSWQ‐C)

The Children’s version of the PSWQ (Chorpita, Tracey, Brown, Collica, & Barlow, 1997) is a 14‐item self‐report measure of general worry. The total score is yielded by summing scores measured on a 4‐point Likert scale, with responses rated from 0 (Never True) to 3 (Always True). Possible scores range from 0 to 42. This measure has been found to have good psychometric properties in clinical samples of children (Pestle, Chorpita, & Schiffman, 2008). In the current sample, the internal consistency was excellent (α = .91).

#### Children’s Response Styles Scale (CRSS)

The CRSS (Ziegert & Kistner, 2002) is a 20‐item self‐report measure, divided into two independent subscales: Rumination (CRSS‐Rumination) and Distraction (CRSS‐Distraction), each containing 10 items. Items are rated on an 11‐point Likert scale from 0 (Never) to 10 (Always), and then, total scores within each subscale are averaged to yield two scores with possible ranges from 0 to 11. In the current sample, internal consistency for the subscales of Distraction and Rumination was good (α = .84 and .80, respectively). The measure’s test–retest reliability and convergent and divergent validity have been supported (Ziegert & Kistner, 2002).

#### Mood and Feelings Questionnaire (MFQ)

The MFQ (Angold, Costello, Messer, & Pickles, 1995) is a 33‐item self‐report measure of low mood experienced over the previous 2 weeks, where responses are rated on a 3‐point Likert‐type, from 0 (Not True) to 2 (True). Total scores can range from 0 to 66. This measure is able to discriminate the presence of major depressive disorder in youth with diverse clinical presentations, using a clinical cut‐off of 29 (Burleson Daviss et al., 2006). Internal consistency was excellent in the current study (α = .95) and the psychometric properties of this measure are good (Burleson Daviss et al., 2006; Bilenberg, Costello, & Wesselhoeft, 2018; Thabrew, Stasiak, Bavin, Frampton, & Merry, 2018).

#### Screen for Children’s Anxiety and Related Emotional Disorders (SCARED)

The SCARED (Birmaher et al., 1999) is a 41‐item self‐report measure of anxiety experienced over the past 3 months. Items are rated on a 3‐point Likert scale. The measure consists of five subscales measuring generalized anxiety, panic, social anxiety, separation anxiety, and school avoidance. Total scores for anxiety can range from 0 to 82. Scores of 25 or greater have been recognized as discriminating the presence of an anxiety disorder (Birmaher et al., 1999). This measure has been recognized as a reliable and valid screening tool for anxiety disorders in youth (Hale, Crocetti, Raaijmakers, & Meeus, 2011). In the current sample, the internal consistency of the total scale was excellent (α = .93).

#### Anxiety Disorders Interview Schedule (ADIS)

Participant diagnoses were determined using the ADIS structured diagnostic interview (Silverman & Albano, 2004). ADIS modules were administered for separation anxiety disorder, social anxiety disorder, specific phobia, generalized anxiety disorder, panic disorder, agoraphobia, persistent depressive disorder, major depressive disorder, and obsessive‐compulsive disorder, attention‐deficit hyperactivity disorder, and post‐traumatic stress disorder.

The young person and a caregiver independently completed ADIS interviews with trained research assistants, who were under the supervision of a clinical psychologist. Primary diagnoses were determined using the composite scoring procedure which considers both caregiver and young person responses. Interviews were audio‐recorded, and inter‐rater reliability was completed for 20% of recordings. Inter‐rater agreement was strong for both diagnosis (κ = .97; *p* < .001) and symptom severity ratings (κ = .88; *p* < .001).

### Procedure

Data from this study were obtained in the baseline assessment for the randomized trial. Informed consent was obtained from caregivers and youth during the CAMHS intake appointment. Consenting young people were emailed a link for an online battery of self‐report questionnaires to complete at home prior to an individual face‐to‐face appointment in which the ADIS was administered. Caregivers also completed the ADIS in an individual face‐to‐face appointment. As this study was completed as part of treatment provision, participants were not paid for their participation.

## Results

### Sample characteristics

Only two of the 114 participants did not have a comorbid diagnosis. On average, participants experienced three comorbid anxiety and/or depressive diagnoses according to the ADIS (*M* = 3.21, *SD* = 1.55, range = 0–7). The most common primary diagnosis was major depressive disorder (46.4%), followed by generalized anxiety disorder (32.1%), social anxiety disorder (10.7%), persistent depressive disorder (7.1%), separation anxiety disorder (1.8%), and specific phobia (0.9%). In addition to anxiety and/or depressive disorder diagnoses, 9.7% of the sample were diagnosed with comorbid OCD, 16.8% with PTSD, 17.7% with ADHD, and 0.9% with oppositional defiant disorder.

### Factor structure

#### Data analysis approach

Confirmatory factor analyses were conducted using the laavan package in R. Multivariate tests indicated non‐normality in the data for kurtosis (Mardia’s coefficient = 44.95, *p* < .05). Mahalanobis *d*‐squared distance indicated 13 multivariate outliers within the sample. With these cases deleted list wise, multivariate tests again indicated non‐normality in the data for kurtosis (Mardia’s coefficient = 22.89, *p* < .05). As such, these cases were retained within the dataset and an MLR estimator was applied in each model to adjust fit for violations of multivariate normality. Model fit was assessed using χ^2^, comparative fit index (CFI ≥ .90), root mean square error of approximation (RMSEA; ≤.08 for acceptable fit, ≤.10 for marginal fit, >.11 for poor fit; Kline, 2005), and the RMSEA test of close fit, in which *p* > .05 indicates close fit to the data. Ninety per cent confidence intervals are reported for RMSEA values. As all χ^2^ statistics were significant, standardized root mean square residual values were not interpreted.

#### Confirmatory factor analysis

For the one‐factor model, all 15 PTQ‐C items were loaded onto the one latent variable, as in Bijttebier et al. (2018), χ^2^(90) = 206.27, *p* < .001, CFI = 0.88, RMSEA = 0.11, 90% CI [0.09, 0.12], RMSEA test of close fit *p* < .001. Results suggested that for the one‐factor model, the hypothesis of both perfect fit and close fit were rejected.

We therefore proceeded to fit a three‐factor model, in which three latent variables were defined: a core characteristics factor, a perceived unproductiveness factor, and a mental capacity factor (see Table 1 for item loadings), as per Bijttebier et al. (2018). For the three‐factor model, χ^2^(87) = 162.88, *p* < .001, CFI = 0.92, RMSEA = 0.087, 90% CI [0.068, 0.107], RMSEA test of close fit *p* = .001. Results suggested that for the three‐factor model, the hypothesis of perfect fit was rejected, as was the hypothesis of close fit. However, the CFI was acceptable and RMSEA was marginal. A chi‐square difference test comparing one‐factor and three‐factor models preferred the three‐factor model, χ^2^(3) = 34.23, *p* < .001.

**Table 1:** Standardized factor loadings for the best fitting three‐factor model

PTQ‐C item	1	2	3
The same thoughts keep going through my mind again and again	.784		
My thoughts come on and I can’t do anything against it	.859		
I can’t stop thinking about it	.862		
I think about many problems without solving any one of them		.809	
I can’t do anything else while thinking about my problems			.819
The same thoughts return into my mind	.750		
Thoughts come into my mind without me wanting them to	.803		
When I am thinking about certain things, I get stuck and find it difficult to stop these thoughts	.771		
I keep asking myself questions without finding an answer		.642	
My thoughts prevent me from focusing my attention on other things			.806
I keep thinking about the same things all the time	.766		
Thoughts just pop into my mind	.672		
I feel as if I must keep thinking about the same things	.570		
My thoughts are not much help to me		.717	
My thoughts take up all my attention			.672

Note

Factor 1 = core characteristics; 2 = unproductiveness; 3 = mental capacity. All items loaded significantly, *p* < .001.

Examination of the modification indices indicated that item 15 appeared as consistently having residual correlations with other items. Two approaches were taken into account for this, allowing residuals to covary between item 15 and items 5 and 10 for which high residual correlations (*r* > .80) were observed and removing item 15 from analysis (Cronbach’s alpha for the scale remained = .94). Model fit was similar between the two approaches. We therefore report the model in which residuals were covaried.

Model fit remained similar for the one‐factor model, χ^2^(88) = 175.66, *p* < .001, CFI = 0.91, RMSEA = 0.09, 90% CI [0.075, 0.11], RMSEA test of close fit *p* < .001, and slightly improved for the three‐factor model, χ^2^(85) = 156.33, *p* < .001, CFI = 0.93, RMSEA = 0.086, 90% CI [0.066, 0.105], RMSEA test of close fit *p* = .002. Again, a chi‐square difference test comparing one‐factor and three‐factor models preferred the three‐factor model, χ^2^(3) = 15.30, *p* = .002. All standardized loadings were strong for this revised three‐factor model (presented in Table 1).

Overall, these results indicate that neither the one‐ nor three‐factor model provided a close fit to data obtained from a clinical sample. Of the two, a three‐factor model (with covaried residuals) was preferred, which provided an acceptable but not good fit to the data.

#### Bifactor model

As a unidimensional model did not provide a strong fit for the data, we next evaluated a bifactor model. The specified model included a general perseverative thinking factor accounting for shared variance among all items, and the three subscale factors which may account for unique variance above and beyond that accounted for by the general factor. Model fit was again borderline, χ^2^(75) = 159.12, *p *< .001, CFI = 0.93, RMSEA = 0.10, 90% CI [0.075, 0.12]. Multidimensionality indices (Rodriguez, Reise, & Haviland, 2016) indicated that estimated common variance for the general factor was high (ECV = 0.82), suggesting that the general factor explained 82% of common variance, with 18% of the variance spread across the subscale factors. The high ECV of the general factor may suggest that a unidimensional model is appropriate for the data (Rogriguez et al., 2016). The percentage of uncontaminated correlations (PUC = 0.60) for the bifactor model suggested that there was likely to be a small difference in the factor loadings between a unidimensional model and the general factor in a bifactor model. In sum, these additional analyses suggest that multidimensionality across the separate subscales is low and that most of the meaningful variance may be better represented by a single factor model of perseverative thinking.

#### Descriptive statistics

Mean score on the PTQ‐C was 43.72 (*SD* = 10.84, range = 2–60), over one standard deviation higher than the mean reported in subclinical samples (*M* = 27.98, *SD* = 12.80; Bijttebier et al., 2015). Means did not significantly differ between males and females, *t*(112) = 0.81, *p* = .42. There was however a difference between those with a primary diagnosis of a depressive (*n* = 62) versus anxiety (*n* = 51) disorder, *t*(82.82) = 1.96, *p* = .053, *d = *0.37, such that those with a primary diagnosis of a depressive disorder demonstrated a higher mean (*M* = 45.84, *SD* = 7.89) than those with a primary diagnosis of an anxiety disorder (*M* = 41.98, *SD* = 12.10). On the separate subscale means, no gender differences were observed, *t*s(112) < 1.59, *p*s > .11. Mean subscale scores did not differ between those with a primary diagnosis of anxiety or depression for the unproductive, *t*(111) = 1.44, *p* = .15, *d = *0.27, or mental capacity subscale, *t*(111) = 1.56, *p* = .12, *d = *0.29, but did for the core characteristics subscale, *t*(80.60) = 2.03, *p* = .046, *d = *0.38, such that those with a primary depressive disorder (*M* = 28.08, *SD* = 4.70) demonstrated higher scores for core characteristics than those with a primary anxiety disorder (*M* = 25.63, *SD* = 7.51).

#### Reliability

Internal consistency was strong for the core characteristics (α = .92) and mental capacity (α = .86) subscales and acceptable for the perceived unproductiveness subscale (α = .77). Internal consistency for the PTQ‐C total score was excellent, α = .94.

#### Validity

Pearson’s correlations between all measures, including the PTQ‐C subscales, are presented in Table 2. Significant, positive correlations of moderate strength between the PTQ‐C total score and subscales, and the content‐specific measures of repetitive negative thinking (PSWQ for worry and CRSS‐Rumination) suggested adequate convergent validity. A weak negative correlation between CRSS‐Distraction and PTQ‐C total score suggested adequate divergent validity with other thinking styles. There was minimal variation in correlation strength between the different PTQ subscales (see Table 2).

**Table 2:** Mean (standard deviation) scores and Pearson correlations between all self‐report measures, and number of diagnoses

		*M* (*SD*)	1	2	3	4	5	6	7	8	9	10	11	12	13	14	15
1	Number of diagnoses	3.21 (1.55)	‐														
2	PTQ‐C	43.72 (10.84)	.43**	‐													
3	PTQ‐core	26.75 (6.62)	.41**	.97**	‐												
4	PTQ – unproductiveness	8.59 (2.47)	.40**	.85**	.76**	‐											
5	PTQ – mental capacity	8.39 (2.72)	.34**	.85**	.74**	.64**	‐										
6	SCARED total	44.87 (14.99)	.59**	.54**	.51**	.49**	.46**	‐									
7	Panic	11.75 (6.07)	.56**	.49**	.45**	.53**	.44**	.87**	‐								
8	General anxiety	13.13 (4.08)	.50**	.57**	.56**	.49**	.45**	.81**	.62**	‐							
9	Social anxiety	9.60 (3.54)	.36**	.24*	.21*	.31**	.16	.63**	.34**	.49**	‐						
10	Separation anxiety	5.74 (3.94)	.38**	.31*	.31**	.25*	.27*	.74**	.57**	.45**	.35**	‐					
11	School anxiety	4.64 (2.14)	.31*	.33**	.29*	.27*	.38**	.57**	.48**	.35**	.25*	.33**	‐				
12	Mood & Feeling Questionnaire	41.67 (15.31)	.38**	.63**	.57**	.58**	.59**	.51**	.54**	.48**	.14	.31**	.32**	‐			
13	Penn State Worry Questionnaire	30.08 (8.19)	.50**	.68**	.70**	.52**	.55**	.68**	.60**	.73**	.33**	.44**	.32**	.46**	‐		
14	CRSS‐Rumination	6.79 (1.70)	.25*	.57**	.55**	.51**	.49**	.41**	.40**	.50**	.14	.16	.24*	.46**	.45**	‐	
15	CRSS‐Distraction	4.38 (1.92)	‐.19*	‐.30*	‐.25*	‐.34**	‐.26*	‐.17	‐.15	‐.17	‐.14	‐.09	‐.15	‐.32**	‐.20*	.01	‐

Note

* indicates significance at *p* < .05; ** indicates significance at *p* ≤ .001 (all two‐tailed). Items 7–11 are subscales on the SCARED.

#### Associations with symptoms

As expected, we observed a significant positive correlation of moderate strength (see Table 2) between the PTQ‐C total score and self‐report measures of anxiety (SCARED) and depression (MFQ), suggesting criterion validity. Again, there was minimal variation in correlation strength between the different PTQ‐C subscales.

We next completed two separate hierarchical regression analyses to determine whether content‐specific measures of repetitive negative thinking accounted for additional variance in depressive (MFQ) and anxiety (SCARED) symptoms, over and above the transdiagnostic PTQ‐C. As number of diagnoses was significantly associated with both anxiety and depression scores, this was entered at step 1 in each analysis, PTQ‐C subscale scores were entered at step 2, and worry (PSWQ) and rumination (CRSS‐Rumination) scores were entered at step 3.

For anxiety , PSWQ did account for additional variance in symptoms, *F* (6, 109) = 23.10, *p* < .001. In step 3 (see Table 3), after PSWQ was added, core characteristics, unproductiveness, and mental capacity no longer accounted for significant variance in anxiety symptoms. Results remained the same when the analysis was repeated with the PTQ‐C total score, β = .05, *p* = .65, *F* (4, 105) = 33.00, *p* < .001. These results suggest that retaining a content‐specific measure of worry may be useful in a clinically anxious group.

**Table 3:** Linear regressions predicting symptom scores

	β	*t*	*p*
Anxiety			
Number of diagnoses	.29	3.84	<.001
Worry (PSWQ)	.50	5.21	<.001
Rumination (CRSS)	.09	1.12	.264
PTQ‐Core	‐.21	‐1.57	.119
PTQ‐Unproductiveness	.16	1.53	.128
PTQ‐Mental Capacity	.10	1.03	.305
Depression			
Number of diagnoses	.11	1.22	.225
Worry (PSWQ)	.04	.349	.728
Rumination (CRSS)	.13	1.43	.155
PTQ‐Core	.04	.276	.783
PTQ‐Unproductiveness	.24	2.01	.046
PTQ‐Mental capacity	.278	2.44	.016

Note

PTQ = Perseverative Thinking Questionnaire; PSWQ = Penn State Worry Questionnaire; CRSS = Children’s Response Styles Scale.

For depression (Table 3), neither PSWQ or CRSS‐Rumination accounted for additional variance in symptoms, *F* (6, 103) = 13.88, *p* < .001. Again, results remained the same when using PTQ‐C total score, PSWQ, β = −.01, *p* = .92, CRSS‐Rumination, β = .14, *p* = .12, *F* (4, 105) = 19.39, *p* < .001, suggesting that content of repetitive negative thinking may not need to be taken into account.

## Discussion

The study represents the first validation of the Perseverative Thinking Questionnaire‐Child version (PTQ‐C) in young people with diagnosed anxiety and depressive disorders. Excellent internal consistency and good convergent and divergent validity were observed for the total PTQ‐C score and all three subscales across the highly comorbid sample. Although a unidimensional three‐factor model of the measure provided the best fit for the data, in bifactor analysis multidimensionality across the separate subscales was low, suggesting that the individual subscales were unlikely to account for substantially greater variance above and beyond that accounted for by a general perseverative thinking factor. We had ample power to reject the hypothesis of perfect fit and an acceptable amount of data for the number of parameters associated with the one‐factor model, and every factor loading was strong (and of a similar magnitude to that observed in the non‐clinical validation) and statistically significant, suggesting that lack of power is not a strong account for the model fit we observed. Overall, the pattern of factor structure results was similar to that found in the subclinical validation of the measure (Bijttebier et al., 2015), although model fit appears poorer in the current clinical sample.

This study offers a number of insights into the use of the PTQ‐C in treatment‐seeking samples. As expected, mean score on the PTQ‐C (43.72) was much higher than that observed in community samples and, notably, was similar for males and females. In terms of transdiagnostic use of the measure, correlations with PTQ‐C subscales and total score were of a similar, moderate magnitude for both depressive and anxiety symptoms, and for the content‐specific worry and rumination measures, although core characteristics scores were higher in those with a primary diagnosis of depressive versus anxiety disorders. Overall, results indicate that a process‐focussed measure of repetitive negative thinking is likely to have utility across clinically anxious and depressive presentations.

Results did indicate that a content‐specific measure of worry accounted for extra variance in anxiety symptoms, suggesting that it may be useful to retain a disorder/content‐specific measure in a clinically anxious group. Neither PTQ‐C subscales nor total score accounted for additional variance in anxiety symptoms once worry was considered, indicating that a process‐only assessment may not comprehensively capture the mechanism associated with clinical anxiety. Clinically, these findings may suggest that the content of worries should be considered when working with young people with multiple anxiety disorders, which is indeed integral to a cognitive‐behavioural treatment approach. As we noted in the Introduction, measures of worry do contain items which are similar to assessment items used to measure symptoms of anxiety (e.g., ‘I’ve been a worrier all my life’ on the PSWQ and ‘I am a worrier’ on the SCARED), and on the SCARED measure of anxiety used in this study, 11 of the 41 items (27%) include the word ‘worry/worried’. Consistent with this, the correlations between the worry measure and anxiety measure were much higher (particularly for the generalized anxiety subscale, which is most strongly characterized by uncontrollable worry) than those between the PTQ‐C subscales and the anxiety measure. This result may therefore more accurately reflect overlap in the items used index worry and anxiety and warrants further longitudinal investigation. For depressive symptoms, the content of repetitive negative thinking appeared not to have such a strong association with depressive symptoms, as the rumination measure did not account for additional variance in symptoms. Interestingly, there is less overlap in the scale items used to index rumination and depression. Our pattern of results is somewhat consistent with Bijttebier et al.’s (2015) initial evaluation of the PTQ‐C and symptoms in a school‐based sample, as the prior study found that content‐specific measures accounted for additional variance over and above the PTQ‐C for both depression and anxiety. Overall, our findings suggest that content‐focussed assessment may still prove useful when working with clinical anxiety; however, overlap in the questions used to index worry and anxiety does need to be considered.

Our conclusions on the role of repetitive thinking in symptoms are limited by our cross‐sectional design, as was our ability to determine the stability of the PTQ‐C over time in a clinical sample. Future longitudinal examination of the measure with larger clinical samples, and also in the context of treatment, may further clarify the underlying factor structure of the PTQ‐C, along with the predictive value of the measure for clinical prognosis. Our results do suggest adequate psychometric properties for use of the measure with treatment‐seeking young people experiencing multiple and comorbid difficulties. As repetitive negative thinking is a key process targeted by transdiagnostic treatments for emotional disturbance, accurate and reliable measurement of this process will be key to future evaluation of the mechanisms underlying transdiagnostic treatment effects.

## Conflicts of interest

All authors declare no conflict of interest.

## Author contribution

**Hitchcock Hitchcock:** Conceptualization (equal); Data curation (equal); Formal analysis (equal); Methodology (equal); Project administration (equal); Resources (equal); Supervision (equal); Writing – original draft (equal); Writing – review & editing (equal). **Renee Brown:** Data curation (equal); Formal analysis (equal); Project administration (equal); Writing – original draft (equal); Writing – review & editing (equal). **Vanessa Cobham:** Conceptualization (equal); Data curation (equal); Funding acquisition (equal); Methodology (equal); Resources (equal); Supervision (equal); Writing – original draft (equal); Writing – review & editing (equal).
